# Diagnostic accuracy of a dynamically increased red blood cell distribution width in very low birth weight infants with serious bacterial infection

**DOI:** 10.1186/s13052-021-00994-w

**Published:** 2021-02-27

**Authors:** Bin-Fang Guo, Su-Zhen Sun

**Affiliations:** 1 Department of Pediatrics, Hebei Medical University, Shijiazhuang, 050000 Hebei China; 2 Department of Pediatrics, Children’s Hospital of Hebei Province, Shijiazhuang, 050031 Hebei China

**Keywords:** Red cell distribution width, Diagnosis, Bacterial infection, Neonatal

## Abstract

**Objective:**

Serious bacterial infection (SBI) remains an important cause of morbidity and mortality in preterm infants. The objective of this study was to evaluate the dynamically increased value of the red cell distribution width (RDW) in the diagnosis of SBI.

**Methods:**

This retrospective study enrolled 334 preterm infants with birth weight less than 1500 g. The initial RDW and the maximum value of RDW during hospitalization were extracted from the MIMIC-III database (version 1.4). Infants were categorized into four groups according to baseline RDW value and ΔRDW (ΔRDW = RDW at maximum- RDW at baseline). Logistic regression analysis was used to assess the risk of developing SBI in each group. A receiver operating characteristic (ROC) curve analysis was used to evaluate the diagnostic value of RDW at baseline alone, ΔRDW alone, and in combination.

**Results:**

Infants with increased RDW at baseline (> 17%) and ΔRDW > 2% exhibited the highest risk of developing SBI, whereas the patients with normal RDW level at baseline (≤ 17%) and ΔRDW≤2% (the reference group) had the lowest risk. This association remained unaltered even after adjustment in multivariable models. Basing on ROC curve analysis, the area under the curve predicted by the combination of RDW at baseline and ΔRDW for SBI was 0.81 (95% CI, 0.76–0.87). Sensitivity and specificity were 78.16 and 72.47% respectively.

**Conclusions:**

We observed that combination of elevated RDW at baseline and dynamic increases during hospitalization is significantly associated with SBI. Therefore, that combination could be a promising independent diagnostic indicator of SBI in newborns.

## Introduction

Infections account for 40% of neonatal deaths worldwide each year [1]. Preterm infants weighing less than 1500 g, called very low birth weight (VLBW) infants, represent a more vulnerable group of newborns. Almost 25% of VLBW infants experience more than one episode of nosocomial infection [2]. Efforts to address neonatal infections are critical to achieving survival goals in newborns [3], early recognition of infection and timely responses are vital to reduce morbidity and mortality among neonates. Currently, in the clinical work, cultures are the gold standard for laboratory diagnosis of bacterial infection even though they lack sensitivity in neonates [4]. Despite serving as predictors of sepsis in neonates, C-reactive protein, IL-6 and procalcitonin have some limitations and are not available in some centers [5].

Previous studies have demonstrated that red blood cell distribution width (RDW) could be a laboratory indicator of infection or inflammation [6]. RDW is a routinely reported hematology parameter as part of the complete blood count [7] which can be automatically measured by modern hematological analyzers. Recently, increasing evidence has proven that RDW may be a frequent predictor for inflammatory diseases in adults, such as pancreatitis and hepatitis [8, 9]. To date, most previous studies that have investigated the relationship between RDW and infection have utilized a single RDW measurement at initial presentation. Recent evidence suggests that RDW can be considered as a dynamic variable with rapid changes associated with acute disease states, and dynamic change of RDW from baseline can provide more prognostic information than the baseline RDW value alone [10, 11]. However, there are few studies reportingthe use of RDW in neonates for monitoring and determining infant’s serious bacterial infection (SBI; including urinary tract infection [UTI], bacterial meningitis, and/or bacteremia).

Also, little is known about the potential impact of changes in RDW from baseline on diagnosis value in infants with SBI. Thus, this study was designed to test the hypothesis that the combination of elevated RDW at baseline and a dynamic increase in RDW from baseline can reflect SBI states and provide more diagnostic information than the baseline or dynamically increased RDW value alone.

## Materials and methods

### Data source

This was a retrospective observational study in which data were extracted from the Medical Information Mart for Intensive Care III (MIMIC-III) database (version 1.4), a comprehensive and free database. MIMIC-III is a public database jointly developed by the Laboratory for Computer Physiology at Massachusetts Institute of Technology (MIT), Beth Israel Deaconess Medical Center, and Philips Healthcare. The database has records regarding the demographics, vital signs, and survival data of nearly forty thousand distinct adult patients and eight thousand neonates who stayed in critical care units between 2001 and 2012 [12]. The MIMIC-III Clinical Database is available on PhysioNet (doi: 10.13026/C2XW26). The Institutional Review Boards (IRB) of MIT approved the creation of MIMIC-III. One of the authors (Bin-Fang Guo, certification number: 36077987) has passed a web-based course on the website of National Institutes of Health (NIH) and was approved for extracting data from MIMIC III for research purpose. Informed consent was waived because all data are from a publicly available database.

### Case inclusion criteria

Newborns who were admitted to the neonatal intensive care unit (NICU) within 24 h after birth were eligible for inclusion in the study. The criteria for exclusion were: (a) birth weight ≥ 1500 g; (b) only one RDW value; (c) congenital disease; (d) multiple gestation; (e) missing gestational age or birth weight or laboratory parameters [including white blood cell (WBC), hemoglobin (Hb), platelet count (PLT)]; (f) red blood cells transfusion.

### Data extraction

Demographic characteristics (gestational age, birth weight, gender), laboratory parameters within the first 24 after entering and the maximum value of RDW in the NICU, total length of hospital stay, and diagnosis (including bacteremia, UTI, bacterial meningitis, preterm infant) were collected, including clinical diagnosis of SBI. The “RDW at baseline” was defined as the initial value of RDW in the NICU. “RDW at maximum” was defined as the maximum value of RDW in the NICU. ΔRDW was calculated as follows: ΔRDW = RDW at maximum- RDW at baseline. The study outcome was defined as SBI.

### Statistical analyses

Descriptive statistics, including medians and ranges for continuous variables and frequencies and proportions for categorical measures, were calculated according to independent and dependent variables. Baseline characteristics of the groups were compared using one-way analysis of variance or Kruskal Wallis test for continuous variables, using chi-square test for categorical variables. The change in RDW between baseline and the maximum value was calculated as ΔRDW. The median value of RDW at baseline was 17% and the median value of ΔRDW was 2%.In addition, infants were categorized into four groups according to baseline RDW value and ΔRDW as follows: group 1, patients with RDW levels in the reference range at baseline≤17% and ΔRDW≤2%; group 2, patients with increased RDW at baseline > 17% and ΔRDW ≤2%; group 3, patients with normal RDW at baseline ≤17% and ΔRDW > 2%; and group 4, patients with increased RDW at baseline > 17% and ΔRDW > 2%. Based on the four groups stratified by baseline RDW value and ΔRDW, the prognostic value of the changes in RDW on SBI was determined using univariate and multivariate logistic regression analysis, adjusted for birth weight, sex, gestational age, WBC, Hb, PLT, which were thought to plausibly interact with both RDW and SBI. Furthermore, receiver operating characteristic (ROC) curves were used to assess the efficacy of RDW at baseline and ΔRDW for the diagnosis of SBI. MedCal software was employed to draw the ROC curve, calculate and compare the area under the curve (AUC). *P* values of less than 0.05 were considered as statistically significant. Statistical analysis was performed using IBM SPSS version 26.0 (IBM, Armonk, NY, USA) and MedCal Vers.15.8 for Windows (MedCalc Software, Ostend, Belgium).

## Results

### Characteristics of infants

**Fig. 1:**
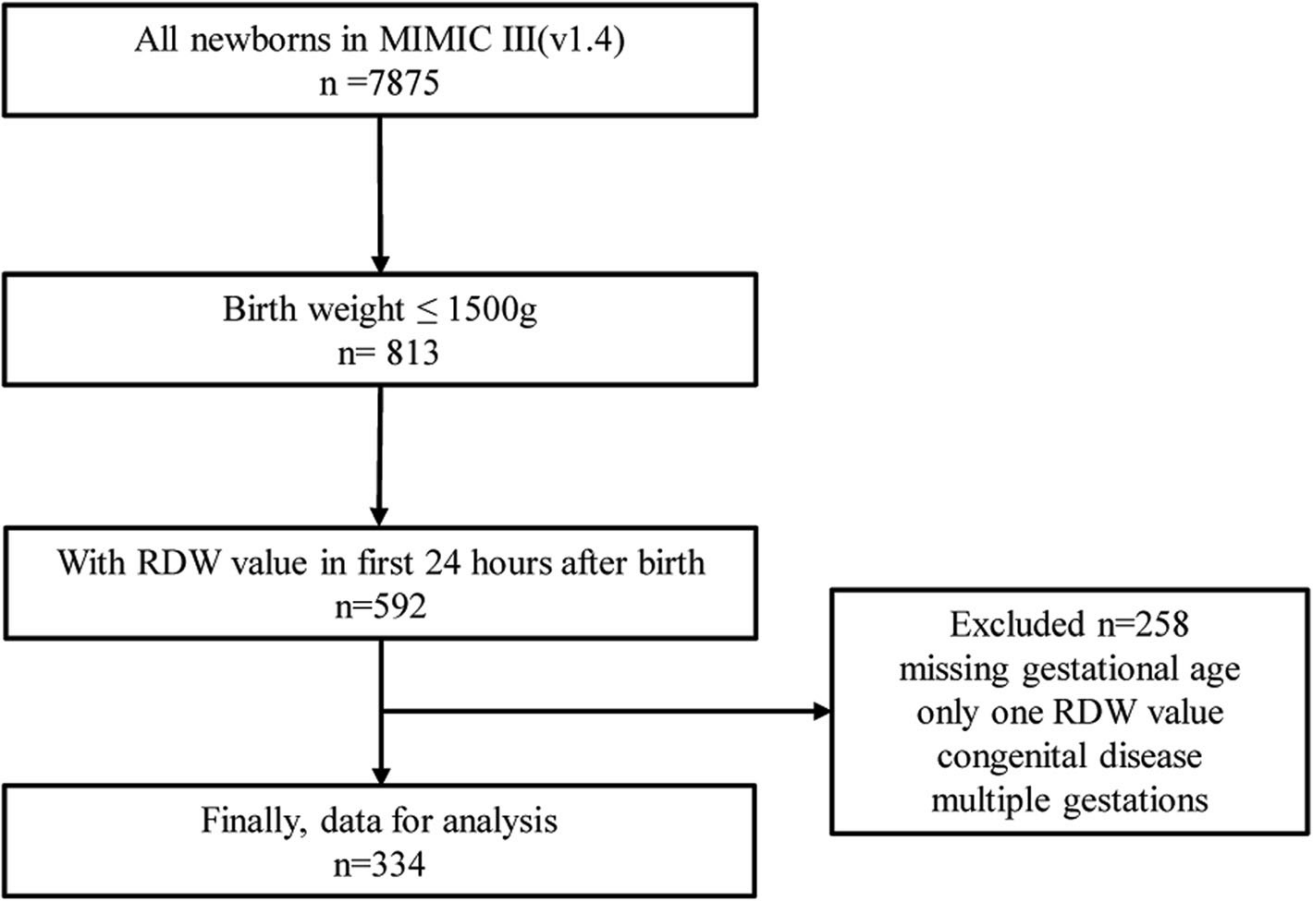
Flow chart for subject identification and inclusion

**Fig. 2:**
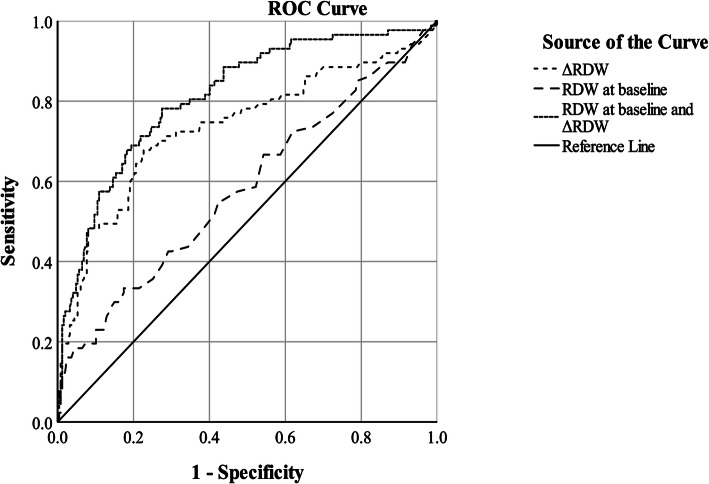
Receiver operating characteristic curves for prediction of SBI by RDW at baseline and ΔRDW

A flow chart summarizing the study selection process is presented in Fig. 1. A total of 334 infants with more than one RDW value were included in the final analyses, and of 87 infants with SBI (75 of bacteremia, 5 of meningitis, 1 of UTI, 10 of bacteremia and meningitis, 2 of bacteremia and UTI). The mean gestational age of the infants was 29.3 weeks. The mean birth weight was 1020 g and 52.4% of patients were male. RDW levels at baseline ranged from 14.1 to 24.2% (median 17%) and ΔRDW ranged from 0.1 to 16.6% (median 2%). Table 1 indicates the baseline demographic and clinical characteristics of each group stratified by baseline RDW value and ΔRDW. There were statistically significant differences in laboratory tests including WBC, Hg, PLT, between the four groups. Compared with the other groups, group 1 exhibited significantly higher birth weight. Group 4 had the highest proportion of patients with SBI (*P* < 0.001) (Fig. 2), and the longest hospitalization (P < 0.001).

**Table 1:** Comparison of clinical, demographic and laboratory characteristics of the study groups

Variables	Group 1 (***n*** = 70)	Group 2 (***n*** = 94)	Group 3 (***n*** = 109)	Group 4 (***n*** = 61)	***P*** value
**Demographic data**					
Gestational age at birth, week median (IQR)	29.20 (26–32.3)	29.5 (28.0–31.5)	28.7 (27–32.3)	28.2 (26.0–32.5)	0.366
Birth weight, g	1.22 (1.06–1.36)	1.10 (0.93–1.32)	0.80 (0.69–1.06)	0.84 (0.70–1.03)	< 0.001
Male, n (%)	39 (55.7)	49 (52.1)	52 (47.7)	35 (57.4)	0.602
Cesarean section delivery, n (%)	52 (74.3)	79 (84.0)	72 (66.1)	50 (82.0)	0.015
**Biochemical data**					
RDW at baseline, % median (IQR)	16.2 (15.7–16.6)	1.7.9 (17.4–19.2)	16.1 (15.7–16.4)	17.8 (17.4–19.0)	< 0.001
RDW at maximum, % median (IQR)	17.15 (16.7–17.7)	18.9 (18.4–19.7)	20.3 (19.2–22.0)	21.6 (20.4–23.4)	< 0.001
△RDW, % median (IQR)	0.1 (0.6–1.6)	0.7 (0.4–1.3)	4.4 (3.1–6.1)	3.1 (2.5–4.5)	< 0.001
WBC, × 10^9^ /L median (IQR)	7.45 (5.2–9.9)	6.8 (4.6–10.1)	7.3 (5.2–9.6)	5.5 (4.1–7.1)	0.039
Hb, g/dL	15.16 ± 0.29	15.68 ± 0.25	14.84 ± 0.19	14.64 ± 0.28	0.017
Neutrophil, %	27.69 ± 1.60	30.06 ± 1.73	29.56 ± 1.48	33.02 ± 2.34	0.295
PLT, 10^9^/L median (IQR)	231 (189–281)	208 (164–282)	233 (199–284)	167 (125–212)	< 0.001
**Severity bacterial infection, n (%)**	3 (4.3)	19 (20.2)	36 (33.0)	29 (47.54)	< 0.001
Sepsis, n (%)	3 (4.3)	17 (18.1)	32 (29.4)	29 (47.54)	
Meningitis*, n (%)	0 (0)	2 (2.1)	8 (7.3)	5 (8.20)	
Urinary tract infection*, n (%)	0 (0)	2 (2.1)	0 (0)	1 (1.64)	
Total length of hospital stays, day	56.6 ± 3.5	50.1 ± 2.9	77.1 ± 4.1	77.7 ± 4.6	< 0.001

RDW at baseline, the initial RDW value at admission; RDW at maximum, maximum RDW value during hospitalization; △RDW = RDW at maximum- RDW at baseline; WBC, white blood cell; Hemoglobin, Hb; PLT, platelet count; IQR, interquartile range. * In group 2, one infant suffered from both meningitis and bacteremia, and one infant suffered from both bacteremia and urinary tract infection. In group 3, four infants suffered from both meningitis and bacteremia. In group 4, five infants suffered from both meningitis and bacteremia, and one infant suffered from both bacteremia and urinary tract infection. Reference group: Group 1.

### An increase in RDW was significantly associated with SBI

Group 1 is the reference category; Table 2 presents the results from univariate and multivariate logistic regression analysis for SBI according to baseline RDW value and ΔRDW. Univariate and multivariate logistic regression analysis showed that groups 2, 3 and 4 had high OR values, which was positively associated with SBI. After adjustment for birth weight, sex, gestational age, WBC, Hb, PLT, OR value was higher in group 4 than in other groups (OR = 10.96, 95% CI: 2.98–40.32, *P* < 0.001).

**Table 2:** Univariate and multivariate logistic regression analysis for SBI according to baseline RDW and ΔRDW

Groups	Univariate logistics analysis		Multivariate logistic regression	
	OR (95%CI)	*P* value	OR (95%CI)	*P* value
Group 1	reference	–	reference	–
Group 2	5.66 (1.60–19.98)	0.007	4.68 (1.31–16.76)	0.018
Group 3	11.01 (3.24–37.44)	< 0.001	5.88 (1.65–20.95)	0.006
Group 4	20.24 (5.74–71.43)	< 0.001	10.96 (2.98–40.32)	< 0.001

CI, confidence interval; multivariate logistic regression analysis, adjusted for birth weight, sex, gestational age, white blood cell, hemoglobin, platelet count.

**Fig. 3:**
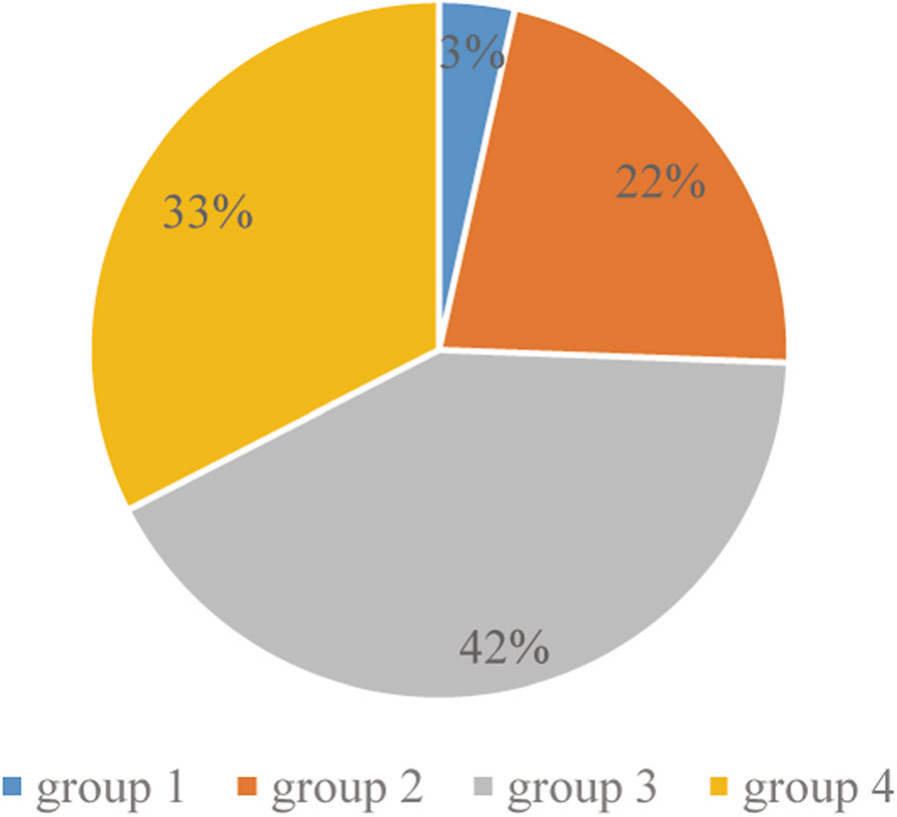
Proportion of each group in SBI

The power of RDW at baseline and ΔRDW for prediction of SBI was demonstrated in Table 3 and Fig. 3. The ROC-AUC was the highest for the combination of RDW at baseline and ΔRDW (0.81), followed by ΔRDW alone for prediction (0.74) (*p* = 0.001).

**Table 3:** Area under receiver operating characteristic curve of RDW

	AUC (95%CI)	*P* value	Sensitivity%	Specificity	+LR	-LR
RDW at baseline	0.58 (0.51–0.66)	0.023	33.33	82.59	1.91	0.81
△RDW	0.74 (0.67–0.805)	< 0.001	66.67	77.33	2.94	0.43
RDW at baseline+△RDW	0.81 (0.76–0.87)	< 0.001	78.16	72.47	2.84	0.33
P* **(AUC)**	0.001					

AUC, area under curve; CI, confidence interval; +LR, positive likelihood ratio; −LR, negative likelihood ratio; P*(AUC). AUC of ΔRDW compared with AUC of RDW at baseline and △RDW.

## Discussion

The present study is a retrospective clinical investigation of the diagnosis value of increased RDW in infants with SBI. This study found that the combination of elevated RDW at baseline and dynamically increased RDW from baseline could reflect infection disease states and provide more diagnostic information than the baseline or dynamically increased RDW value alone. Specifically, patients in group 4, who had increased RDW at baseline (RDW > 17%) and dynamic increase during hospitalization (ΔRDW > 2%), exhibited the highest risk of SBI, whereas patients in groups 2 and 3, who had increased RDW level at baseline (RDW > 17%) or ΔRDW> 2%, had a lower risk of SBI. Group 1 served as the reference. This significant association between an increase in RDW and SBI remained unaltered even after adjusting for various confounding variables.

In our study, the comparison of predictive accuracy of baseline RDW, ΔRDW, and the combination of the two showed that the combination parameter had superior performance in prediction of SBI. This finding was consistent with a recent study, which showed a significant increase in RDW levels in infants with gram-negative sepsis. A RDW cut-off of > 19.50% was associated with prediction of late-onset Gram-negative sepsis (*P* < 0.001), with a sensitivity of 87% and a specificity of 81% [13]. Another study reported that high RDW became a risk indicator for critical newborns [14]. Moreover, one study reported that increased RDW values were associated with the severity of sepsis in neonates [15]. However, Ju XF et al. showed that continuous increase in RDW level, rather than the level of RDW at baseline, was more beneficial in predicting in-hospital death of elderly patients with septic shock [16]. Our present study found that not only elevated baseline RDW but also increased ΔRDW were associated with SBI. In clinical settings, elevated baseline RDW in preterm infants is caused by or associated with intrauterine infection, which is a major cause of premature delivery [17]. Despite the lack of obvious symptoms of system infection, preterm infants are more susceptible to serious infections [18]. It is vital to initiate timely antimicrobial therapy during a bacterial infection episode, therefore we should clarify the noble prediction value of the RDW during the entire hospital stay.

The reason why patients with SBI have a higher RDW remains poorly understood. Some potential mechanisms by which infection causes RDW elevation have been reported. RDW represents the size variance in circulating erythrocytes, so in any physiologic process that upregulates erythropoiesis or causes an increased release of immature red block cells into circulation, RDW becomes elevated [19, 20]. One study found that RDW could measure the efficiency of biological control, therefore, it may be a predictor of the function of organism [21]. Inflammation not only disrupts the survival of erythrocytes but also deforms red block cell membranes [22, 23]. Taken together, increased systemic inflammation is the major theorized mechanism that results in an increase in RDW [23, 24].

To the best of our knowledge, this study is the first to report that the combination of an increase in RDW at baseline and dynamic increase during hospitalization plays a potential role in predicting newborns developing SBI. However, this study has several limitations. First, we arbitrarily determined the median of RDW value as a measurement and defined ΔRDW as an increase in RDW. It remains unclear whether changes in RDW during hospital stay could represent the pathophysiologic changes. The range of RDW value in normal or pathological conditions has not yet been determined [6], and increased RDW indicates that the inflammatory system is active in patients [12]. Therefore, we investigated the clinical outcomes of the patients with SBI by the RDW at baseline and ΔRDW. Second, this study is conducted base on a public database, therefore, it is unknown whether use of erythropoietin, iron or vitamin B12, and reticulocyte count, could have affected RDW values. Moreover, RDW is a red blood cell index that is rapidly and automatically calculated by all modern hematological analyzers such as Sysmex XE-2100 analyzer, Sysmex-XT-2000i counter (Sysmex, Kobe, Japan), and ADVIA 2120i instrument (Siemens, Munich, Germany) [13, 25, 26]. The reference interval of RDW varies with the instrumental used [25, 26]. The information of the hematological analyzers was not recorded in MIMIC-III, different instruments and measurement techniques used to obtain RDW might have limited interpretation and direct application of the results in other medical institutions. Third, standard deviation RDW (RDW-SD) was not considered in this study. Finally, this is an observational study, which may have a bias or a lack of randomly distributed exposure, and confusion causality. Further research should be undertaken to investigate the predictive value of RDW at baseline and dynamic change.

## Conclusions

Our results imply that an increase in RDW from baseline through the hospitalization is significantly associated with SBI. Therefore, a combination of an increased baseline RDW value and a dynamically increased RDW could be a promising independent diagnostic indicator in infants with SBI. This study provides support for future investigations considering changes of RDW and the associated stratification of critically ill infants at risk for infection.

## Data Availability

The generated data sets are available from the corresponding author on reasonable request.

## Abbreviations

SBI: Serious bacterial infection
RDW: red cell distribution width
ΔRDW: ΔRDW = RDW at maximum- RDW at baseline
VLBW: very low birth weight
MIMIC-III: Medical Information Mart for Intensive Care III
ROC: receiver operating characteristic
AUC: area under the curve
VLBW: very low birth weight
UTI: urinary tract infection
IRB: Institutional Review Boards
NIH: National Institutes of Health
NICU: neonatal intensive care unit
WBC: white blood cell
PLT: platelet count
